# Patient-derived organoids as a potential model to predict response to PD-1/PD-L1 checkpoint inhibitors

**DOI:** 10.1038/s41416-019-0616-1

**Published:** 2019-10-31

**Authors:** Giosue Scognamiglio, Annarosaria De Chiara, Antonina Parafioriti, Elisabetta Armiraglio, Flavio Fazioli, Michele Gallo, Laura Aversa, Rosa Camerlingo, Francesco Cacciatore, Gianluca Colella, Roberto Pili, Filomena de Nigris

**Affiliations:** 1 Pathology Unit, Istituto Nazionale Tumori - IRCCS - Fondazione G. Pascale, Naples, Italy; 2 Department of Pathology, ASST Centro Specialistico Ortopedico Traumatologico Gaetano Del Pini - CTO, Milan, Italy; 3 Division of Musculoskeletal Oncology Surgery, Istituto Nazionale Tumori- IRCCS - Fondazione G. Pascale, Naples, Italy; 4 Department of Cell Biology and Biotherapy, Istituto Nazionale Tumori - IRCCS - Fondazione G. Pascale, Naples, Italy; 5 Department of Translational Medical Sciences, Federico II University, Naples, Italy; 6 Department of Hematology/Oncology, Indiana University Medical School, Indianapolis, IN USA; 7 Department of Precision Medicine, University of Campania L. Vanvitelli, Naples, Italy

**Keywords:** Cancer, Molecular medicine, Drug discovery

## Abstract

Selection of cancer patients for treatment with immune checkpoint inhibitors remains a challenge due to tumour heterogeneity and variable biomarker detection. PD-L1 expression in 24 surgical chordoma specimen was determined immunohistochemically with antibodies 28-8 and E1L3N. The ability of patient-derived organoids to detect treatment effects of nivolumab was explored by quantitative and qualitative immunofluorescence and FACS analysis. The more sensitive antibody, E1L3N (ROC = 0.896, *p* = 0.001), was associated with greater tumour diameters (*p* = 0.014) and detected both tumour cells and infiltrating lymphocytes in 54% of patients, but only 1–15% of their cells. Organoids generated from PD-L1-positive patients contained both tumour cells and PD-1/CD8-positive lymphocytes and responded to nivolumab treatment with marked dose-dependent diameter reductions of up to 50% and increased cell death in both PD-L1-positive and negative organoids. Patient-derived organoids may be valuable to predict individual responses to immunotherapy even in patients with low or no immunohistochemical PD-L1 expression.

## Background

Immunotherapies targeting the programmed cell death-1 receptor (PD-1) and its ligand-1 (PD-L1) yielded impressive clinical results in advanced cancers expressing high levels of PD-L1.^1,2^ However, novel treatments for rare cancers are limited by insufficient patients and trials to establish treatment benefits. This is particularly true for chordomas, rare malignant tumours predominantly located in the spinal axis with a high local recurrence rate (43–85%) and a low tendency for distant metastasis.^3^ Chordomas are resistant to chemotherapy (standard treatment: surgery and carbon ion**–**radiotherapy^4^), and are candidates for immunotherapy because they express more PD-1/PD-L1 than healthy bone tissues.^5–7^ Clinical trials evaluated the efficacy of targeting the PD-L1 axis with nivolumab alone or in combination with ipilimumab^8^ and a trial combining nivolumab with stereotactic radiosurgery is ongoing (NCT02989636), but no data have yet been published on the correlation of PD-L1 expression and outcomes. In terms of clinical strategy, a selection of cancers/patients sensitive to anti-PD-L1 blockage therapy on the basis of PD-L1 expression in their tumour cells and infiltrating lymphocytes would be highly desirable. Here, we compared PD-L1 recognition by two antibodies, separately assessing expression in tumour cells and tumour-infiltrating lymphocytes of whole surgical specimens, and correlated results with clinical parameters. Because the potential advantages of organoids over cancer cell cultures are increasingly recognised,^9,10^ we also generated patient-derived organoids and determined the dose-dependent effects of nivolumab by quantifying diameters, apoptosis, and PD-L1 expression, to establish the potential of this approach for the prediction of treatment responses.

## Methods

### Patients

Twenty four primary chordoma patients treated at the G. Pascale Institute and the G. Pini Institute. Sections of surgical specimens were stained with two monoclonal antibodies to PD-L1, E1L3N and 28-8 (Cell Marque) according to manufacturer’s instructions, using BenchMark XT kits and an automatic immunostainer (Ventana Medical Systems). PD-L1-positive cancer cells and lymphocytes were determined as percentages of positive cells in all section,^11^ conforming to FDA guidelines.

Flow cytometry and detailed Methods**:** See Supplement.

### Organoid cultures, treatments and immunofluorescence

Fresh tissues were digested in 1 mg/ml collagenase type-I and 0.05% trypsin for 45 min. 10^3^ cells/well were seeded in 2% matrigel (R&D System)-coated microchambers (μ-Slide 3D #5816, Ibidi) and incubated in RPMI with 10% FBS for 72 h. Different concentrations of nivolumab (Bristol-Myers Squibb) or control IgG were added to the media for another 24 h. Organoids were fixed and incubated with specific antibodies for 1 h (see Supplement for details). Fluorescence imaging was performed on a LSM 700 confocal microscope (Zeiss, Germany). Twenty six layers (z-projection) of each image were scanned and analysed with FV10-ASW 4.2 software. Results were reported as percentages of DAPI-stained cells. Organoid diameters were determined by ImageJ (National Institutes of Health, Bethesda, MD). Statistical analyses were performed with SPSS v21.0 (Chicago, IL, USA). Significance was set at *p* < 0.05.

## Results

The baseline characteristics of patients are shown in (Supplementary Table 1). The median age of patients was 65 years (range 55–79). The mean follow-up period was 73.0 months, with a minimum of 6 months. At the time of analysis, 3 (12%) patients had died, and median overall survival was 50 months (95% CI: 63.8–98.6).

**Fig. 1:**
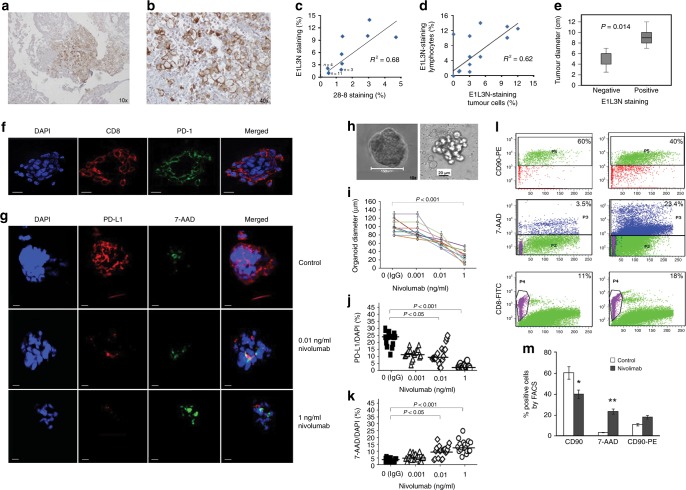
Results of immunohistochemistry and organoid experiments. **a**, **b** Immunostaining of PD-L1 in chordoma sections. Representative section of a chordoma immunostained with the more sensitive monoclonal antibody, E1L3N, using a BenchMark XT kit. Original magnification: ×10 **a**, ×40 **b**. **c** Linear regression analysis of the percentages of tumour cells stained by antibodies E1L3N and 28-8 in all 24 patients. R^2^ = 0.68 and beta = 0.105. **d** Linear regression between percentage of tumour cells and lymphocytes staining positive to E1L3N antibody (R^2^ = 0.62). **e** Box plot indicating that PD-L1-negative tumours had smaller median diameters than PD-L1-positive ones (6 ± 3 cm vs. 9 ± 6 cm; *p* = 0.014 by Spearman correlation). **f**–**m** Organoids as a model to predict the effect of chordoma treatment. **f** Confocal microscopy images of an untreated organoid generated by growing cells isolated from a fresh surgical chordoma specimen grown for 72 h in matrigel and stained with DAPI (blue), an antibody to CD8+ lymphocytes (red) and to PD-1 (green). The right panel merges the two previous images with a DAPI-stained image indicating organoid cells. Scale bars = 20 μm. **g** TOP: Control organoid treated for 24 h with isotype-matched nonspecific IgG and stained with an antibody to PD-L1 (red) or 7-AAD, a marker of cell death (green). The merged image superimposes the two preceding images with a DAPI-staining. MIDDLE**:** Organoid treated for 24 h with 0.01 ng/ml nivolumab. BOTTOM**:** Organoid treated for 24 h with 1 ng/ml nivolumab. Images shown are representative of 20 organoids in each treatment group, for three patients. Scale bars = 20 μm. **h** Representative light-microscopic images of organoids used to determine their diameters by computer-assisted image analysis (Image J, NIH, Bethesda). Left: control IgG treated organoid Right: 0.01 ng/ml nivolumab treatment. Scale bars: 150 μm and 20 μm respectively. **i** Diameter of organoids in the three nivolumab-treated groups. Diameters reported are mean ± SD from eight different organoids for each dosage (triplicate measurements in three patients). Significance levels were determined by one-way ANOVA. **j** PD-L1 expression in control organoids and organoids treated with the indicated doses of nivolumab. Immunofluorescent cells were determined in confocal images of organoids scanned in 26 layers (z-projection). Data are representative of 20 organoids per each treatment group, from three different patients. Results are reported as percentage of DAPI-stained cells (significance levels indicated were determined by one-way ANOVA. **k** 7-AAD-positive organoid cells reported as a percentage of DAPI-staining cells, as a measure of cell death. **l** FACS-characterisation of control and 1 ng/ml nivolumab-treated organoids pooled from the same patient. Organoid cells were separated by trypsin and at least 10.000 cells were counted. Representative analysis of three independent experiments are shown. TOP: isotype control and setting. MIDDLE: 7-AAD-positive cells. BOTTOM**:** CD8+ lymphocytes. **m** Bar graph summarising quantitative results of all FACS analyses. Data are expressed as the mean ± SD of three independent patient-derived pool organoids. *p < 0.05, **p < 0.001. Significance levels were determined by Student’s *t*-test. *p* < 0.05 was considered significant. FACS Fluorescence-assisted cell sorting*,* PD-1 programmed death 1, PD-L1 programmed death ligand 1

Serial sections of chordoma surgical specimens were assessed with two antibodies, E1L3N and 28-8 (Supplementary Fig. 1 and Supplementary Tables 1–3). PD-L1-positive tumour cells ranged from 1–15% of all tumour cells per section and were mainly localised at the aggressive margin of the tumour (Fig. 1a, b). Staining with both antibodies was correlated by linear regression (R^2^ = 0.68, beta = 0.105) (Fig. 1c), but sensitivity and specificity analysed by ROC statistics was higher for E1L3N than 28.8 (*p* = 0.001) (Supplementary Fig. 1C). This was most evident in samples with low intensity of PD-L1 staining (*p* = 0.001). E1L3N yielded several distinct staining patterns (Supplementary Fig. 2). PD-L1-positive tumour cells detected by E1L3N correlated with positive tumour-infiltrating lymphocytes (R^2^ = 0.62 *p* = 0.001) (Fig. 1d; Supplementary Fig. 3 and Supplementary Tables 2, 3), and with larger tumour sizes (*p* = 0.014) (Fig. 1e and Supplementary Table 1), but not with overall survival or recurrence rate.

Cells of fresh biopsies isolated by collagenase digestion and analysed by FACS confirmed the percentage of PD-L-1 positivity (Supplementary Fig. 4). Biopsy cells were then cultured on matrigel for 72 h, generating organoids with diameters of 80–200 μm (Supplementary Fig. 5A, B). Individual organoids contained both CD8 and PD-1-expressing cells (Fig. 1f). PD-L1-positive cells determined by confocal immunofluorescence (up to 20%) varied considerably (Supplementary Fig. 5C–F), However, 60% of organoids obtained from the biopsy of a PD-L1-positive patient were negative for PD-L1 (Supplementary Fig. 5D), consistent with the immunohistochemical observation that only some tumour cells express PD-L1.

To assess treatment effects, organoids were incubated for 24 h with 0.001, 0.01, or 1 ng/ml of nivolumab, using isotype-matched IgG for controls (Fig. 1g). Treatment effects were assessed by their diameters, PD-L1 expression, and percentages of DAPI-stained cells (Fig. 1h–k). At a dose of 0.01 ng/ml nivolumab, the median organoid diameter was reduced to 70 μm (50% less than control, *p* < 0.01), and at a dose of 1 ng/ml to 40 μm (*p* < 0.001 vs. control) (Fig. 1h, i). At the highest dose, PD-L1 expression was <1% and cell death reached 15% (*p* < 0.001 vs. control) (Fig. 1j, k). FACS analysis of cells isolated from pooled treated organoids from PD-L1-positive patients showed that 1 ng/ml nivolumab reduced CD90-positive cells by 20% and increased cell death to 23% (Fig. 1l, m). In contrast, nivolumab increased relative CD8+ lymphocyte content to 18%, vs. 11% in controls (Fig. 1l, m).

## Discussion

The present results confirm the limitations of detecting PD-L1 by immunohistochemistry to select patient sensitive to nivolumab treatment. Comparison of the two antibodies indicated that E1L3N, the more sensitive one, detected PD-L1 expression in only 54% of spinal chordomas. This is less than the 68.5% reported with a different antibody in tissue arrays,^5^ possibly as a result of different chordoma stages or aggressiveness. Tumour sizes were greater in PD-L1-positive patients and its expression in tumour cells correlated with expression in infiltrating lymphocytes.^5,6^ This is of clinical interest, but does not provide prognostic information. Our results are consistent with those of clinical trials reporting that PD-L1 alone is of limited use to predict response to treatment of chordomas in individual patients. The efficacy of immunotherapy and lower adverse effects than standard treatments has encouraged cancer trials in unselected populations with highly metastatic cancer sarcoma subtypes.^12^

Three-dimensional cell culture are revolutionising the study of human diseases and cancer by permitting analysis of patient-derived tissue with non-invasive procedures.^9,10^ The present results provide the first evidence that patient-derived chordoma organoids allow to test individual responses to treatment. Hundreds of organoids may be generated from fresh tissue to provide a reasonable approximation of tumour heterogeneity.^10^ Pools generated from PD-L1-positive patients containing both PD-L1-positive and negative organoids responded to nivolumab with a significant dose-dependent size reduction within 24 h. This further supports the observation that some sarcomas with low or no immunohistochemically detectable PD-L1 expression respond to therapy.

Limitations of the study include the possibility that the original tumour microenvironment may not have been maintained, and that only a few fresh biopsies were available, due to the rarity of chordomas. Nevertheless, results support the notion that patient-derived spheroids may help to identify subsets of chordoma patients who are likely to respond to immunotherapies, and to compare individual sensitivity to various treatments. They may thus constitute a valuable step towards individually targeted treatment of chordomas and other malignancies.

## Supplementary information

Supplement methods and legends

## Data Availability

All data supporting the study are available on request. No proprietary materials except patient tissues were used.
