# Supplementary material for: Patient-derived organoids as a potential model to predict response to PD-1/PD-L1 checkpoint inhibitors
Source: Br J Cancer. 2019 Oct 31;121(11):979–82. doi: 10.1038/s41416-019-0616-1 (PMC6889147; doi:10.1038/s41416-019-0616-1)

**Electronic Supplement for TH-2019-4161R1**

**Patient-derived organoids as a potential model to predict response to PD-1/PD-L1 checkpoint inhibitors**

Giosue Scognamiglio, Annarosaria De Chiara, Gaetana Parafioriti, Elisabetta Armiraglio, Flavio Fazioli, Michele Gallo, Laura Aversa, Rosa Camerlingo, Francesco Cacciatore, Gianluca Colella, Roberto Pili, Filomena de Nigris

**methods**

Patients

The study retrospectively analysed 24 specimens from patients who were diagnosed with primary chordoma in the sacrum or spine at the G. Pascale Institute of Naples and the G. Pini Institute of Milan and were surgically resected. None of the patients had chemotherapy, radiation, or other chordoma treatment before surgery. Histologic subtypes were determined according to the WHO classifications. The diagnoses were confirmed by at least two pathologists and the stage was defined according to the American Joint Committee on Cancer staging system. Events were defined as the first evidence of local recurrence or death from any cause, and overall survival was determined at the time of the last clinical follow-up (October 2018). The study was performed in accordance with the Declaration of Helsinki and was a part of the BioBank project of the G. Pascale Institute approved on January 20, 2016 (delibera n.15), and written informed consent was obtained from all participants.

Immunohistochemistry (IHC)

IHC was performed on whole sections, using an automatic immunostainer (BenchMark XT, Ventana Medical Systems, Tucson, AZ, USA) according to the manufacturer’s instructions. Primary monoclonal antibodies against PD-L1 (E1L3N and 28-8 from Cell Marque, UK) were used at a 1:100 dilution. A formalin-fixed, paraffin-embedded tissue sample of each patient was cut into 4 µm thick sections, transferred to poly-l-lysine-coated adhesive slides and dried at 60°C for 1 h. A cell-conditioning solution 1 (CC1) standard (Tris/borate/EDTA buffer, pH 8.4) was used for antigen retrieval, followed by blocking with inhibitor D (3% H_2_O_2_) at 37°C for 4 min. Slides were then incubated with the primary antibodies at 37°C for 60 min. The staining was optimized using a positive and negative control for each marker. Primary antibodies bound were visualized with an UltraView Universal DAB detection kit (Ventana Medical Systems). Immunoreactivity was independently assessed by two pathologists, and discrepancies resolved by joint re-evaluation. Immune checkpoint proteins can be expressed in tumour cells and/or tumour-infiltrating lymphocytes. Lymphocytes were identified as small mononuclear cells with typically scanty cytoplasm. The scoring system adopted was the same as in our previous osteosarcoma study [20]. The stained tissues were scored on a four point scale. Intense membrane staining was scored as 3, moderate membrane staining as 2, light staining as 1, and no staining as 0. PD-L1-positive tumour cells were also determined as a percentage of all tumour cells in the section. H-scores providing a combined measure of staining intensity and percentage of positive cells were calculated as: H = 1 x (percentage of cells staining at intensity 1) + 2 x (percentage of cells staining at intensity 2) + 3 x (percentage of cells staining at intensity 3). Tumour-infiltrating lymphocytes (TILs) were identified by hematoxylin/eosin, and staining for PD-L1 was graded as 0 (absent or rare) or 1 (positive), and as the percentage of positive TILs.

Primary cell cultures

Primary cells derived from patients were freshly isolated as previously described [20]. Briefly, tissue was processed by enzymatic digestion in 1 mg/ml collagenase type-I (Thermo Fisher) and 0.05% trypsin, 0.02% EDTA (Biowest, Nuaille, France~~,~~) for 45 min. Digested tissue was then filtered with 70mm cell strainers, centrifuged, and plated in RPMI 1640 medium (Gibco, Grand Island, NY, USA) supplemented with 10% fetal bovine serum (FBS; Gibco) and antibiotics (100 units/ml penicillin and 100 μg/ml streptomycin). All cell lines were cultured at 37°C in a humidified atmosphere with 5% CO_2_.

Flow cytometry

To evaluate the phenotype of the chordoma, on the day of surgery the expression of the following markers was determined by flow cytometry: mouse anti-human CD90 FITC, mouse anti-human-CD274 PE (PD-L1), mouse anti-human 279 PE-Cy7 (PD-1), mouse anti-human CD44 APC and H-CAM, (clone IgG2b IM7), and FITC anti-human CD45 (clone IgG1 HI30) (all from Miltenyi Biotec, Calderara di Reno, Bologna, Italy). An 7-AAD FITC (BD Biosciences, NJ, USA) was used to determine cell death according to the manufacturer’s instructions. Different organoid pools were harvested with 0.25% trypsin and washed with PBS. After centrifugation, cells were resuspended in 100 μl buffer and then stained with 3 μl of 7-AAD, anti-CD8 (BW135/8) or anti-PD-1 (CD279) (Miltenyi Biotec), incubating the mixture in the dark at 4°C for 15 min. Experiments were repeated three times. After incubation, samples were washed in PBS and analysed by FACS ARIA III (Becton Dickinson). A total of at least 10,000 events were recorded and analysed with DIVA Software (version 6.1.1; BD Biosciences). Background fluorescence was estimated by substituting the primary antibody with isotype-matched controls.

Organoid cultures, treatments and immunofluorescence (IF)

1×10^3^ cells/well from three different biopsies were separately seeded in 2% Matrigel (R&D System) -coated micro chambers (μ-Slide 3D perfusion, Ibidi) in RPMI with 10% FBS. Cells grown on matrigel maintain sphere-forming capacity, and after 72 h yield organoids of 80-150 μm in diameter. Different concentrations of nivolumab ([Bristol-Myers Squibb](https://packageinserts.bms.com/pi/pi_opdivo.pdf)) or control IgG were added to the media for another 24h. Organoids were then fixed for 10 min with 4% paraformaldehyde at room temperature, permeabilized with 0.1% Triton X-100 (Sigma-Aldrich) in PBS with 1% bovine serum albumin, blocked in 10% goat serum, and incubated for 1 h at room temperature in 1% goat serum with either E1LN3, Anti-PD-1 antibody (ab52587, Abcam) or 7-AAD, a maker of cell death (A1310, Thermo Fisher). When required, primary antibodies were incubated for 30 min with Alexa-conjugated secondary 593 or 488 (Thermo Fisher). Nuclei were labelled with 49,6-diamidino-2-phenylindole (Sigma-Aldrich). The percentage of staining cells was determined by counting at high magnification (100x). For each treatment group, images of 20 organoids were captured, 26 layers (z-projection) of each image were scanned, and results were reported as a percentage of all DAPI-stained cells. Fluorescence imaging was performed on a Zeiss LSM 700 confocal microscope (Jena, Germany) equipped with FV10-ASW 4.2 software. Organoid diameters were determined by ImageJ (National Institutes of Health, Bethesda, MD).

Statistical analysis

Descriptive statistics are reported as proportions and medians. Categorical variables were evaluated by χ^2^ test, Fisher’s exact test, or Pearson correlation (R) test, as appropriate. Correlation between the two antibodies was assessed by Cohen’s test, and sensitivity and specificity of antibody E1L3N tested by ROC correlation. Data were censored if patients were free of recurrence or alive at the last follow-up. All tests were two-sided, and statistical significance was set at p < 0.05. All statistical analyses were performed using SPSS for Windows (version 21.0, SPSS, Chicago, IL, USA).

**SUPPLEMENTARY FIGURES**

**Supplementary Figure 1. Immunostaining patterns of PD-L1 in chordoma sections.** Representative serial sections of the same chordoma biopsy immunostained with monoclonal antibodies 28-8; **A** and E1L3N **B**, using the 28-8 antibody pharmDx kit on the BenchMark XT, Ventana platform, and the E1L3N kit Dako BenchMark XT, Ventana, at the recommended dilutions and staining times. Original magnification: 63x**.** **C.** ROC curve determining specificity and sensitivity of E1L3N vs. 28-8. The area under the curve was 0.896 for E1L3N and 0.712 for 28-8;

p=0.001.


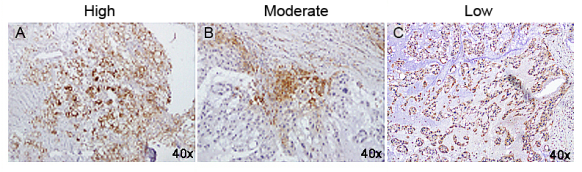


**Supplementary Figure 2. PD-L1 staining patterns of chordoma sections using the E1L3N antibody. A.** Intense PD-L1 staining pattern, mainly in the cell membranes of neoplastic cells; **B.** Moderate PD-L1 membrane staining of the section of another patient, mainly localized at the tumour; **C**. Low-intensity pattern lacking distinct staining of tumour cell membranes. Original magnifications are indicated in the figure.

**Supplementary Figure 3. PD-L1 expression in tumour cells and tumour-infiltrating lymphocytes. A:** Representative images of PD-L1 recognized by antibody E1L3N in tumour cells and **B** in immune cells of the same chordoma sample. Original magnification: 63x.


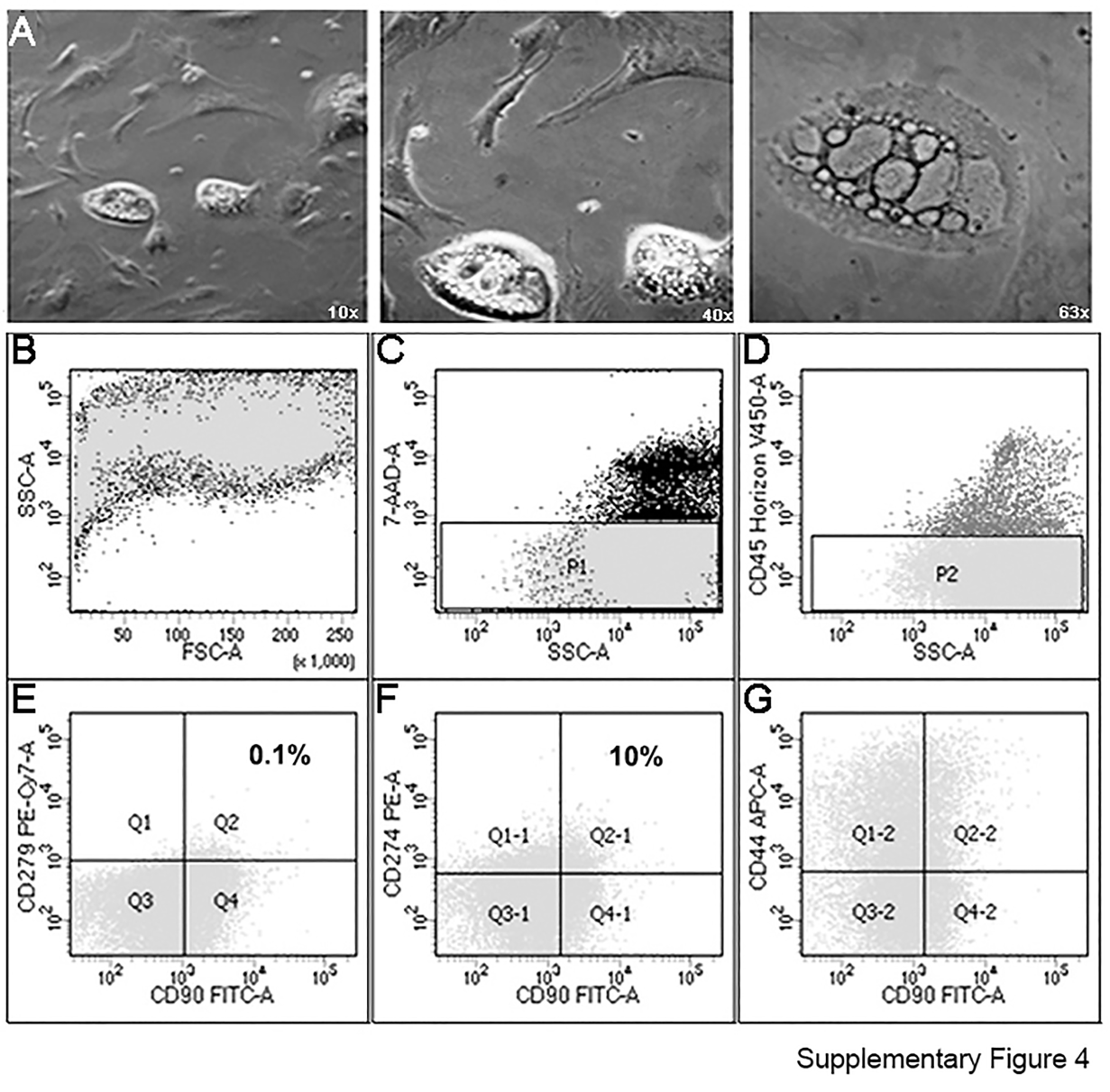


**Supplementary Figure 4. PD-L1 and cell markers on the surface of cells isolated from chordoma biopsies determined by FACS. A:** Optical microscope images of biopsy isolated cells at different magnifications. **B, C.** Cell sorting controls. **D**. Representative cells from chordoma biopsy sorted with CD45 antibody. **E**. Cells from chordoma biopsy sorted with CD279 (PD-1) and CD90 antibodies showing that 1% of cells are double positive. **F**. Cells from chordoma biopsy sorted with CD274 (PD-L1) and CD90 antibodies, identifying 10% of double positive cells. **G**. Cells sorted with CD44 and CD90 antibodies.


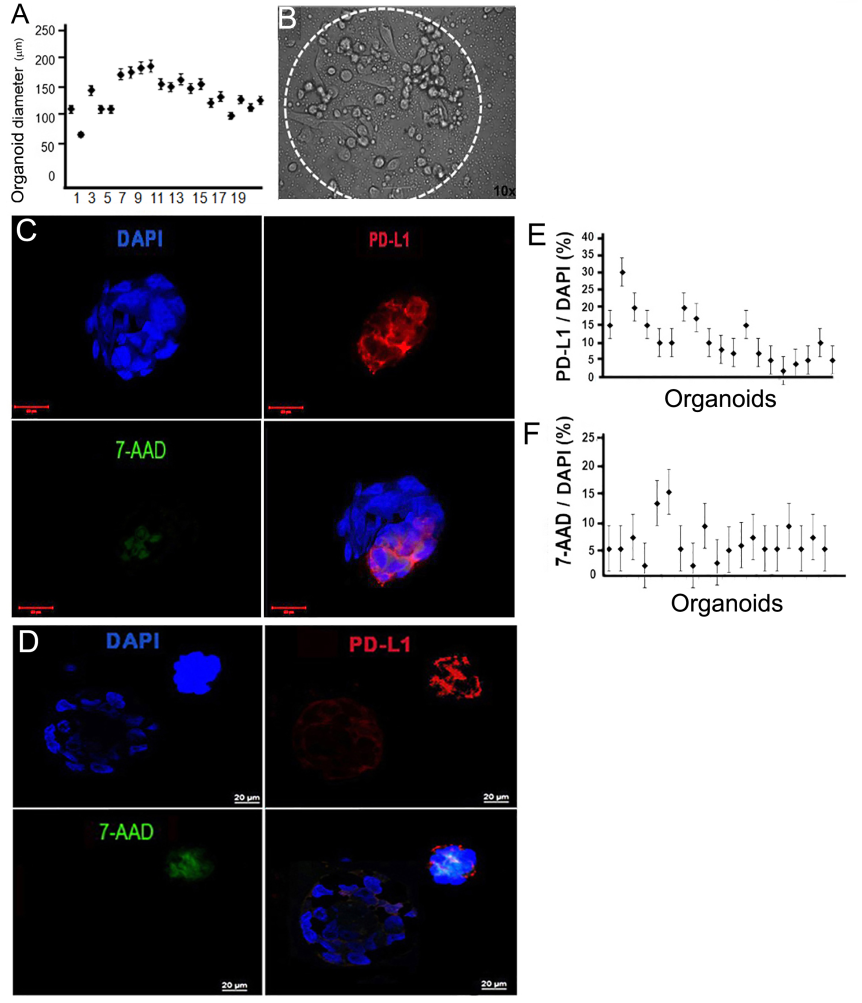


**Supplementary Figure 5.** **Organoids as a model to predict the effect of chordoma treatment. A.** Diameters of 20 different organoids after 3 days (mean ± SD). **C.** Representative optical image of an organoid section showing cells with different morphology. Original magnification: 10x. **D.** Representative confocal microscopy images of an organoid stained with DAPI, the E1L3N antibody to PD-L1, and 7-AAD, as well as an overlay image. Scale bars: 20 μm. **E.** Representative images of two organoids from the same patient, one PD-L1 positive and one PD-L1 negative, after 24h treatment with 1ng/ml of nivolumab, stained for DAPI, PD-L1 and 7-AAD expressing cells (plus merged image). **F,G.** Apoptotic and PD-L1-expressing cells were determined in confocal images of organoids scanned in 26 layers (z-projection), and results are reported as a percentage of the cells staining with DAPI. The median of apoptotic cells was 7%, and PD-L1 positive cells was 15%. Significance levels indicated were determined by one-way ANOVA. Data are representative of 20 organoids per each treatment group, in three different patients, and triplicate experiments.

**Supplementary Table 1** Patient characteristics by PD-L1 expression in tumour and immune cells by antibody E1L3N

| **Characteristic** | **Study**  **cohort** | **PD-L1 in tumour (E1L3N)**  **Positive Negative** | | **Significance**  **(P value)** |
| --- | --- | --- | --- | --- |
| Patients (n) | 24 | 13 (54.2%) | 11 (45.8%) |  |
| Mean age (years)  (range 55-79) | 64.8 | 64 ± 10 | 65.7 ± 1 |  |
| Males  Females | 19  5 | 9 (47.4%)  4 (80.0%) | 10 (56.6%)  1 (20%) | 0.59 |
| Median tumour diameter (range: 4-15 cm) | 7 ± 4.5 | 9 ± 6 | 6 ± 6 | **0.014** |
| Tumour location:  Sacral vertebra  Cervical, thoracic, or lumbar | 14 (58%)  10 (41%) | 5 (20%)  7 (29%) | 9 (37.5%)  3 (12.5%) | 0.58  0.24 |
| Monocytes *  < 8 x 10^8^/L  ≥ 8 x 10^8^/L | 7 (29%)  17 (71%) | 4 (16%)  7 (29%) | 3 (12.6%)  10 (41%) | 0.89  0.78 |
| Leukocytes *  < 7.5 x 10^9^/L  > 7.5 x 10^9^/L | 9 (38%)  15 (62%) | 4 (16%)  8 (33%) | 5 (20%)  7 (29%) | 0.78  0.98 |
| Extent of TILs | 24 | 12 (50%) | 12 (50%) | 0.68 |
| Post-operative recurrences  Mean time (months) | 7  40 | 4 (53.3%)  36 | 3 (42.9%)  38 | 0.46 |
| Overall survival (months)  (Follow-up range: 6-150 mo  mean=73) | 50  95% CI: 63.8-98.6 | 48 | 52 | 0.80 |

* in peripheral blood

**Supplementary Table 2.** Global positive percentage of agreement between IHC with antibody E1L3N in tumour cells (T) and tumour infiltrating lymphocytes (I) and Pearson test.


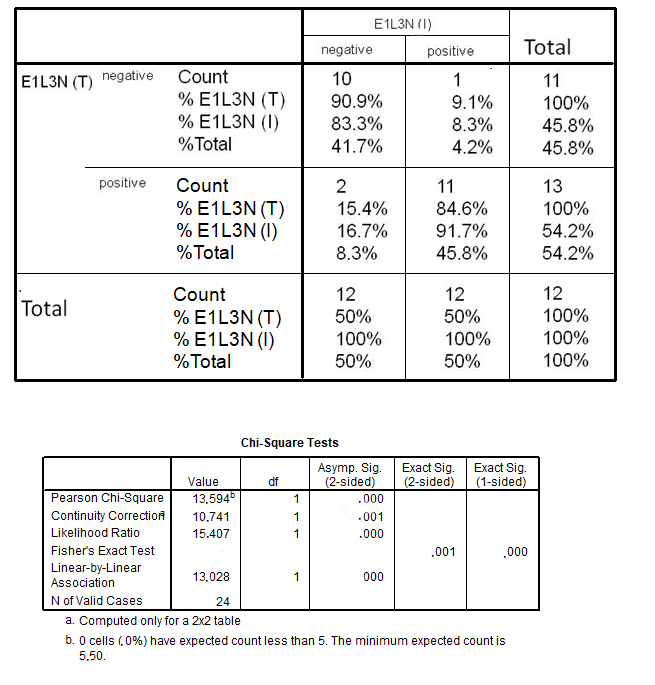


24

24

**Supplementary Table 3.** Global positive percentage of agreement between IHC with antibody 28-8 in tumour cells (T) and tumour infiltrating lymphocytes (I).


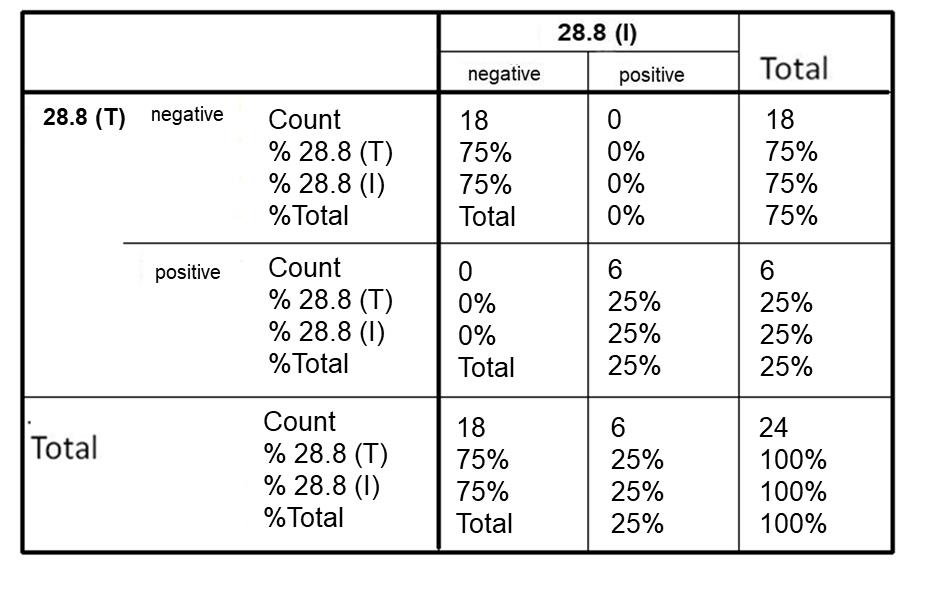

Supplement: Supplementary file 1 — Supplement methods and legends [file 41416_2019_616_MOESM1_ESM.docx]
